# Progress in Bacteriology

**Published:** 1901-10-05

**Authors:** 


### PROGRESS IN BACTERIOLOGY.

**Human and Bovine Tuberculosis.**—The subject of the relationship between human and bovine tuberculosis occupied much attention at the Tuberculosis Congress recently held in London. Experimenting with cattle Koch[1] found that pure cultures of human tubercle bacilli, or the sputum of phthisical patients, were non-infective to these beasts when introduced by inhalation, by feeding, by intraperitoneal or intravenous injection. On the other hand when the experiments were made with bovine tubercle bacilli the infection was speedy, intense, and rapidly fatal. Swine fed with food impregnated with human phthisical sputum were found at the expiration of three and a half months to present no lesions beyond little nodules in the lymphatic glands of the neck and a few grey nodules in the lungs. On the other hand when bovine tubercle bacilli were employed, without exception the lesions were severe, and in half the cases terminated fatally. Similar results followed intravascular inoculation into asses, sheep, and goats. From these facts Koch concludes that probably man is insusceptible to bovine tuberculosis, though the proof of this is difficult of accomplishment. Undoubtedly cases of primary abdominal tuberculosis are rare, when one considers the large amount of tuberculous material ingested. McFadyean[2] was opposed to Koch's views. He pointed out that tuberculin produced a specific reaction in tuberculous cattle whether human or bovine bacilli had been employed in its preparation. The difference between human and bovine tubercle bacilli was one of intensity of virulence only, and generally all pathogenic bacteria common to all the domestic animals were pathogenic to man. Primary abdominal infection was not as rare as was generally accepted, but reached from 28 to 29 per cent. Although 30 per cent. of the milch cows of this country were tuberculous, he thought only about 2 per cent. had the udder infected and were capable of disseminating the disease by the milk. Ravenal, from a series of comparative experiments, concluded that the bovine tubercle bacillus has a high degree of pathogenic power for man also which is especially manifest in the early years of life. Moeller mentioned that mammalian tuberculosis could be modified by passage through pigs, and he himself had so modified the tubercle bacillus by passage through the slowworm that even after passage through a warm-blooded animal it would not grow above 30° C. Hueppe[3] recalls that Koch formerly regarded avian and mammalian tubercle bacilli as different organisms, a theory that has since been controverted by the experiments of Fischer, Nocard and others. He admits that the bovine tubercle bacillus has peculiar characteristics and most closely resembles the bacillus of leprosy, but this he attributes to parasitic adaptation. All varieties of human tubercle bacilli behave alike when injected into small animals, and the failure with large animals is probably due to insufficient dosage. Hueppe mentions, on Heller's authority, that tabes mesenterica constitutes 50 per cent. of the tuberculous affections of children. Replying to Hueppe, Baumgarten[4] mentions that in 1893 he injected two calves, the one with bovine and the other with human tubercle bacilli, and that whilst the former succumbed to general tuberculosis the latter exhibited no evil effects. He also relates that Rokitansky in the hope of effecting a cure in inoperable cases of cancer injected six persons suffering from this disease with cultures of bovine tubercle. Although many survived for some weeks none of the six cases showed at any time evidences of tuberculosis nor were any such signs found at the post-mortem examinations. Whilst he believes in the identity of the human and bovine tubercle bacillus, the latter has, in his opinion, undergone such modification as to render it harmless to man. Klein[5] favours the identity of the human and bovine bacilli. Seven per cent. of samples of country milk contained tubercle bacilli. He mentions that the inoculation test is the only satisfactory method of examination, and notes that old non-pathogenic glycerine-agar cultures of the tubercle bacillus rapidly become virulent if transferred to milk. The pseudo-tubercle bacillus has such characteristic methods of growth that it can rarely be confused with the real organism.

**Bacteriology of Milk and Butter.**—Bergey[6] found cocci in 90 per cent. of samples of market milk, and streptococci in 50 per cent. Streptococci are, however, seldom found in samples taken from the best dairies; in the present investigation only three times in 59 examinations. The streptococcus pyogenes aureus is often present in milk, and may be highly virulent. Streptococci in milk may determine severe gastro-intestinal disorder in children. Klein[7] has isolated a special streptococcus from milk which he names the streptococcus radiatus (pyogenes), from its characteristic growth on gelatine. Out of a large series of milks tested, the pseudo-tubercle bacillus was present in 8 per cent., in one case the diphtheria bacillus was found and proved virulent. In another sample of milk an organism was isolated, which from its resemblance to the Klebs Löffler organism was named the bacterium diphtheroides. Amongst other varieties commonly present in milk are B. lactis, proteus vulgaris, B. coli, B. mesentericus, and B. enteritidis. Pathogenic yeasts are also occasionally present. Tobler[8] examined twelve samples of butter, using guinea-pigs for inoculation purposes. In two cases the tubercular lesions were produced and from these the tubercle bacillus isolated. Five samples gave negative results, and the remaining five were found to contain acid-fast bacilli resembling tubercle bacilli, but failing to produce the typical lesions in inoculated animals.

[1] Brit. Med. Jour., July 27, 1901. [2] Ibid., Aug. 3, 1901. [3] Berl. Klin. Woch., Aug. 2, 1901. [4] Ibid., Sept. 2, 1901. [5] Jour. of Hyg., vol. i., p. 78, 1901. [6] Amer. Med., April 20, 1901. [7] Med. Chron., vol. i., 1901. [8] Zeit. f. Hyg., vol. xxxvi., p. 121.

